# Faisabilité et pertinence de la check-list de sécurité au bloc opératoire central du Centre Hospitalier Régional de Saint Louis du Sénégal

**DOI:** 10.11604/pamj.2017.28.96.11428

**Published:** 2017-09-29

**Authors:** Moustapha Diedhiou, Philippe Manyacka, Mactar Dieng, Jacques Noel Tendeng, Mohamed Lamine Diao, Ousmane Thiam, Hady Tall, Issa Thiam, Ibrahima Konaté

**Affiliations:** 1 Service d’Anesthésie-Réanimation, Centre Hospitalier Régional de Saint Louis, Sénégal; 2 Service de Chirurgie Générale, Centre Hospitalier Régional de Saint Louis, Sénégal; 3 Service de Gynéco-obstétrique, Centre Hospitalier Régional de Saint Louis, Sénégal; 4 Service d’Oto-rhino Laryngologie, Centre Hospitalier Régional de Saint Louis, Sénégal; 5 Service d’Urologie, Centre Hospitalier Régional de Saint Louis, Sénégal

**Keywords:** Check-list, sécurité bloc opératoire, hôpital, Checklist, security, operating block, Hospital

## Abstract

**Introduction:**

La check-list (CL) de sécurité au bloc opératoire est un outil de qualité qui permet de réduire la morbimortalité péri opératoire. C’est une des exigences de la Haute Autorité de Santé (HAS) française dans la procédure de certification des établissements de santé. L’objectif de ce travail est de réaliser une évaluation quantitative et qualitative de l’utilisation de cet outil au sein du bloc opératoire central du centre hospitalier Régional de Saint Louis.

**Méthodes:**

Une évaluation prospective des indicateurs de l’utilisation pratique de la check-list et de son apport dans l’amélioration des pratiques chirurgicales au bloc opératoire du centre hospitalier régional de Saint-Louis a été entamée depuis son lancement en mars 2016.

**Résultats:**

Le taux d’utilisation de la CL était de 75%, le taux de conformité était de 60%; le taux de renseignements était de 99% pour les items pré-induction, 93% pour les items pré-incision et 88% pour les items postopératoires. Seules 73% des CL analysées avaient été remplies avec une communication orale effective aux trois temps. La CL a permis de détecter des dysfonctionnements matériels et/ou des événements indésirables dans 15% des cas. Aucune erreur d’identification de patient ni de coté opéré ne furent objectivées dans notre étude.

**Conclusion:**

La CL participe au développement de la culture de sécurité dans les blocs opératoires, et a débouché sur la mise en place d’une cartographie des risques au bloc opératoire. Certes importante, elle ne doit néanmoins pas être présentée comme l’outil magique pour éviter les erreurs, mais s’intègre dans l’amélioration de la qualité des soins avec d’autres programmes comme le signalement d’évènements indésirables et les revues de morbi-mortalités.

## Abstract (en)

**Introduction:**

Safety checklist (CL) in the operating block is a quality tool which allows to reduce perioperative morbidity and mortality. That is one of the French National Health Authority requirements for the certification procedure of healthcare institutions. This study aimed to conduct a quantitative and qualitative evaluation of the use of this tool in the central operating block at the Regional Medical Center in Saint Louis.

**Methods:**

A prospective evaluation of the indicators for monitoring the practical use of the checklist and the contribution to the improvement of surgical practices in the operating block at the Regional Medical Center in Saint Louis was initiated since the launch of this study in March 2016.

**Results:**

CL utilization rate was 75%, compliance rate was 60%; information rate was 99% for the pre-induction items, 93% for the pre-incision items and 88% for the postoperative items. Only 73% of analyzed CL were filled with an effective oral communication according to the three items. CL helped to detect hardware failures and/or adverse events in 15% of cases. No patient’s identification or operated side error were objectified in our study.

**Conclusion:**

CL contributes in the development of the culture of patient’s safety in the operating block and has led to the establishment of a risk mapping in the operating block. Nevertheless, while important, it shouldn’t be considered a magic tool to avoid errors but integrated into the improvement in health care quality with other programs such as the reporting of adverse events and the the review of morbidity and mortality.

## Introduction

La check-list (CL) de sécurité au bloc opératoire est un outil de qualité qui permet de réduire la morbimortalité péri opératoire [1]. Formellement recommandée par l’Organisation mondiale de la santé (OMS), elle a montré sa capacité à réduire la mortalité périopératoire de 1,5 à 0,8% et le taux de complications de 11 à 7% dans une étude internationale multicentrique portant sur plus de 8 000 patients [2]. C’est une des exigences de la Haute Autorité de santé (HAS) française dans le cadre de la démarche de certification des établissements de santé. Cette check-list OMS adoptée par la HAS de France comporte trois parties: une avant l’induction anesthésique, une avant l’incision chirurgicale et une dernière après l’intervention [3] (Annexe 1). Elle n’est néanmoins pas d’utilisation courante au Sénégal. Le centre hospitalier régional de Saint Louis du Sénégal est un hôpital médicochirurgical avec une capacité litière de 240 lits; Il y existe pratiquement toutes les spécialités chirurgicales basiques qui s’épanouissent dans un bloc opératoire où sont réalisés environ 2000 actes anesthésiques par an soit 40 actes opératoires par semaine. Dans le contexte de développement et de diversification des actes anesthésiques et chirurgicaux et sous l’initiative du coordonateur du bloc opératoire, la check-list de sécurité fut instaurée conformément à la recommandation de l’OMS. L’objectif principal de cette étude prospective a été d’y évaluer la bonne utilisation de la check-list OMS après formation du personnel à son utilisation. L’objectif secondaire a été de déterminer l’impact de l’utilisation de cet outil dans l’amélioration des conditions de travail pour le personnel et des conditions sécuritaires pour le patient.

## Méthodes

L’étude a été réalisée à l’hôpital régional de Saint Louis du Sénégal. Le Centre Hospitalier Régional (CHR) de Saint louis est doté d’une capacité litière de 240 lits; et pratiquement toutes les spécialités chirurgicales (hormis la neurochirurgie et la chirurgie cardiovasculaire) y existent et s’épanouissent dans un bloc opératoire doté de 4 salles d’opération. Le personnel soignant du bloc opératoire est composé d’un infirmier anesthésiste et de deux infirmiers instrumentistes par salle d’opération; ils exercent sous la responsabilité respective de deux médecins anesthésistes réanimateurs et de neuf chirurgiens toutes spécialités confondues. En mars 2016, sous l’initiative du coordonnateur du bloc opératoire et avec l’adhésion et la collaboration de toute l’équipe, la check-list de sécurité fut insaturée conformément à la recommandation de l’OMS (Annexe 1). Les patients admis au bloc opératoire de mars à septembre 2016 ont été inclus dans notre étude. Les indicateurs d’utilisation pratique de la check-list de sécurité ont été analysés en l’occurrence le taux d’utilisation, le taux de renseignement des différents items, le taux de conformité ainsi que l’impact de la check-list de sécurité dans la détection du dysfonctionnement matériel préalable et des événements indésirables. Pour tous nos patients cette check-list de sécurité s’est déroulée selon un mode de communication orale effective aux trois temps (avant induction - avant incision - après intervention chirurgicale).

## Résultats

**Figure 1:**
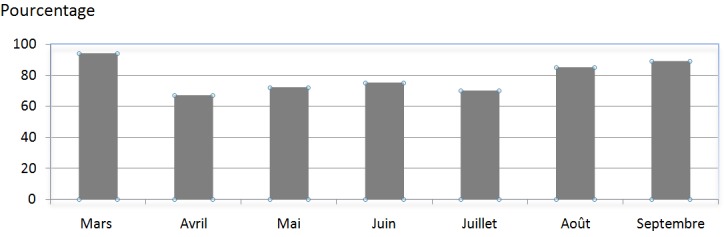
Évolution du taux d’utilisation de la check-list au cours de l’année 2016

Au total tous les 632 patients admis au bloc opératoire du 1er mars au 30 septembre 2016 et ont été inclus dans notre étude. Quatre cent soixante quatorze (474) check-lists ont été remplies et analysées soit un taux d’utilisation ou de réalisation de 75%; le taux de conformité ou de complétude était de 60%, soit pour 284 patients. Seuls 12 patients sur 34 admis au bloc pour une chirurgie en urgence ont bénéficié d’une utilisation de la check-list, soit un taux d’utilisation de 35% pour les chirurgies en urgence. Par ailleurs, 97% des check-lists remplies ont concerné les patients reçus pour une chirurgie programmée. Le taux de remplissage était de 93% pour les zones pré-induction, 99% pour les zones pré-incision et 88% pour les zones post opératoires. Les résultats concernant les indices d’utilisation pratique de la check-list sont présentés dans le Tableau 1. Le taux d’utilisation de la check-list était de 94% pour le mois de mars, 67% pour le mois d’avril, 72% en Mai, 75% en Juin, 70% pour le mois de Juillet, 85% en Août et 89% pour le mois de Septembre (Figure 1). La check-list a permis de déceler un déroulement optimal de l’intervention chirurgical dans 85% des cas et dans 15% des cas elle a abouti à la détection d’événements indésirables et/ou de dysfonctions de matériels avant l’induction anesthésique (Tableau 2).

**Tableau 1:** Analyse pratique de la qualité de remplissage des check-lists

Paramètres	N	%
check-lists analysées	474	75
check-lists non remplies	158	25
Check-lists intégralement remplies	284	60
Check-lists non intégralement remplies	190	40
chirurgies en urgence réalisées	34	-
Check-lists remplies pour les chirurgies en urgence	12	2,5
Check-lists remplies pour les chirurgies programmées	462	97
Zones avant induction remplies	469	99
Zones avant incision remplies	441	93
Zones après intervention remplies	417	88

**Tableau 2:** Apport de la check-list dans la détection d’événements indésirables et des dysfonctions de matériels

Dysfonctions de matériels		Evénements indésirables	
Types	N (%)	Types	N (%)
Bistouri électrique non fonctionnel	6 (1,2)	Non-respect du délai d’antibioprophylaxie	4 (0,8)
Boite de chirurgie non adaptée	2 (0,4)	Critères d’intubation difficile	2 (0,4)
Problème du matériel anesthésique	0 (0)	Erreur d’identification de patient	0 (0)
		Erreur de côté opéré	0 (0)

## Discussion

L’initiative « une chirurgie sûre sauve des vies » a été établie par « l’alliance mondiale pour la sécurité des patients » et fait partie intégrante de la campagne de l’OMS pour réduire le nombre de décès chirurgicaux à travers le monde. L’objectif de cette initiative est d’améliorer la politique et l’organisation clinique des soins pour travailler sur des objectifs de sécurité importants comme les pratiques d’anesthésie inadéquates, les infections chirurgicales évitables et les communications insuffisantes à l’intérieur de l’équipe chirurgicale. Ces dysfonctionnements ont été trouvés fréquents, de gravité extrême mais évitables quels que soient les pays et les organisations [4]. Sous la coordination d’un chirurgien américain, des experts du monde entier représentant les différents métiers et disciplines concernés par le bloc opératoire ont travaillé pour dégager dix objectifs majeurs pour sécuriser les procédures chirurgicales. Ce travail a permis d’établir des recommandations de bonnes pratiques se traduisant par un ensemble de standards sécurité devant être vérifiés avant, pendant et après toute intervention chirurgicale. Dans cette même optique, l’OMS a proposé un support d’implantation de ce programme pour l’amélioration de la sécurité au bloc opératoire à travers l’instauration d’une check-list de sécurité (Annexe 1). Cet outil obligatoire et recommandé formellement par l’organisation internationale est simple et facile à mettre en œuvre dans la pratique [1].

Nous avons ainsi réalisé dans cette même perspective cette étude sur la faisabilité et la pertinence de la check-list de sécurité à l’hôpital régional de Saint Louis, Sénégal. Le principal intérêt est de montrer que la qualité du renseignement de cet outil, initialement médiocre est rapidement améliorée par une mesure simple de sensibilisation du personnel dans ce pays en voie de développement. Dans notre étude, le taux de remplissage est passé de 67% en avril à 89% au mois de septembre. Une tendance similaire a été retrouvée dans l’étude Djiboutienne de Becret A. et al qui ont réussi à imprégner à leur équipe l’intérêt de l’utilisation de cet outil [3]. La première période de notre travail (notamment d’Avril à Juillet) a montré de très mauvais résultats en termes de qualité de remplissage de la check-list, malgré son statut obligatoire. Dans une étude récente, des centres français avaient obtenu des taux de réalisation de la check-list de 95,5% et de 95,8%, mais les taux de complétudes étaient de 64% et 68% [5] sensiblement égaux au taux de complétude de notre étude qui est de 60%. Les mauvais résultats initiaux de notre étude peuvent cependant s’expliquer par deux particularités de notre hôpital: la présence d’infirmiers de bloc qui y travaillent que depuis peu (six mois) et qui ont un nombre important de nouvelles informations à intégrer; la présence d’infirmiers instrumentistes qui ne travaillaient pas auparavant au bloc opératoire mais dans des services cliniques, et n’avaient ainsi jamais été sensibilisé sur l’intérêt d’un tel outil.

Cela montre l’intérêt d’une information régulière de tous les personnels sur le bien-fondé de cette check-list. Cette information doit bien sûr viser les personnels nouvellement arrivés mais aussi ceux qui travaillent depuis plus longtemps dans l’institution et qui pourraient être démotivés par le caractère répétitif de cette check-list. La formation de tout le personnel est une étape primordiale de la mise en place de la check-list dans un bloc opératoire. Globalement, notre étude, menée sur sept mois montre que les indicateurs de pratique de la check-list (taux d’utilisation, taux de renseignement) restent assez stables avec une marge de progression certaine. Les résultats concernant les indicateurs de pratique sont assez proches de ceux rapportés dans les expériences de Nice et de Nancy-Metz [6]. Le défaut de temps pour le remplissage de la check-list est exceptionnel dans notre série. Cette situation concernait surtout les chirurgies en urgence qui ont bénéficié d’un remplissage de la check-list que dans 2,5% des cas. Cette difficulté pour les chirurgies urgentes même si elle est semblable à celle retrouvée dans l’étude de Becret A. et al [3] s’explique par le caractère urgent et par la réduction de l’effectif durant ces actes réalisés en urgence. Une donnée importante à prendre en compte est le fait que la check-list est très rapide à être rempli, et plus les personnes ont l’habitude de la remplir, moins la vérification est longue. Dans notre étude l’effectif réduit ne permet pas d’évaluer l’impact de l’utilisation de la check-list sur la morbi-mortalité mais elle a néanmoins permis de mettre en évidence des dysfonctionnements matériels et des événements indésirables dans 15% des cas (Tableau 2). Ce taux est légèrement plus important que ceux retrouvés dans la littérature justifiant la nécessité d’une continuation et d’une amélioration de l’emploi de cet outil dans notre centre. Le Tableau 3 compare nos résultats à ceux de différentes séries de la littérature. L’absence de compréhension du bien-fondé de la démarche (Check-list), l’indifférence chirurgicale sont des freins au développement de la CL [7]. L’amélioration des indicateurs pratique de la check-list de sécurité en dépit d’une adhésion des professionnels au concept dans notre étude nécessite à l’instar des travaux de T. Gueguen et al une amélioration de l’information et la formation des professionnels travaillant quotidiennement avec cet outil…[8]. Il est sans doute impératif de prendre conscience que ce travail collaboratif (check-list) est garant du développement et de la pérennisation d’une réelle culture de la sécurité et de la qualité des soins. L’absence d’investissement et de soutien dans ces démarches expose les établissements à payer les coûts de la non-qualité. A l’heure où les sources de financement de première ligne des institutions sanitaires en Afrique se font rares, il semble important de rappeler que ces démarches telles que l’utilisation de la check-list sont une aubaine en termes médico-économiques.

**Tableau 3:** Analyse comparative de notre série et celles de la littérature

Etudes(auteur, année)	Taux d’utilisation(%)	Taux de conformité(%)	Dysfonctions de matériels et/ouEvènements indésirables (%)
F Rateau et al. [7]. 2011	73	60	10
C. Paugam et al. [8] 2011	84	92	3
T. Gueguen et al. [8] 2011	50	20	2
P Haynes et al. [2] 2009	73	63	--
Notre étude 2016	75	60	15

## Conclusion

La qualité de l’utilisation de la check-list au bloc opératoire du centre hospitalier régional de Saint Louis (Sénégal) a été grandement améliorée par des mesures simples de formation et d’information du personnel. Sa mise en place parait donc facilement réalisable dans les blocs opératoires d’un pays comme le Sénégal. La check-list participe au développement de la culture de sécurité dans les blocs opératoires et a abouti à la mise en place d’une cartographie des risques au bloc opératoire. Elle est certes un élément indispensable à l’accréditation des établissements sanitaires; mais ne doit néanmoins pas être présentée comme l’outil magique pour éviter les erreurs en salles d’opération. Toutefois, elle doit s’intégrer dans l’amélioration de la qualité des soins avec d’autres programmes tels que le signalement d’évènements indésirables et les revues morbi-mortalités.

### Etat des connaissances actuelle sur le sujet

- L’usage de la check-list de sécurité au bloc opératoire est une recommandation formelle de l’OMS;
- La check-list de sécurité est un outil de qualité qui a permis de réduire la morbi-mortalité péri opératoire;
- La check-list de sécurité n’est pas d’utilisation courante dans les blocs opératoires des pays en voie de développement.

### Contribution de notre étude à la connaissance

- La qualité de l’utilisation de la check-list au bloc opératoire du Centre Hospitalier Régional de Saint Louis (Sénégal) a été grandement améliorée par des mesures simples de formation et d’information du personnel;
- La check-list de sécurité a permis de réduire l’incidence des événements indésirables au sein de notre bloc opératoire;
- Sa mise en place parait facilement réalisable dans les blocs opératoires des pays en voie de développement.

## Conflits d’intérêts

Les auteurs ne déclarent aucun conflit d'intérêt.

## Annexe

**Annexe 1**: check-list de sécurité au bloc opératoire OMS [3]
